# Supplementary material for: Prognosis Following Sustained Virologic Response in Korean Chronic Hepatitis C Patients Treated with Sofosbuvir-Based Treatment: Data from a Multicenter Prospective Observational Study up to 7 Years
Source: Medicina (Kaunas). 2024 Jul 14;60(7):1132. doi: 10.3390/medicina60071132 (PMC11279039; doi:10.3390/medicina60071132)
Supplement: Supplementary file 1 [file medicina-60-01132-s001.zip › medicina-3098178-supplementary.pdf]

# Supplementary Materials：Prognosis following Sustained Virologic Response in Korean Chronic Hepatitis C Patients Treated with Sofosbuvir-Based Treatment: Data from a Multicenter Prospective Observational Study Up to 7 Years

Supplementary Table S1. Characteristics of the recurred or re-infected patients

| Patient | Gender | Age | Genotype | Treatment | Cirrhosis | Alcohol | Time after SVR (months) | HCV RNA (IU/mL) | Child-Pugh | MELD | APRI | FIB-4 |
|---|---|---|---|---|---|---|---|---|---|---|---|---|
| 1 | Male | 42 | 2a | SOF+RBV | + | + | 31 | 80,800 | 7 | 13 | 1.214 | 4.780 |

SVR, sustained virologic response; HCV, hepatitis C virus; MELD, model for end-stage liver disease; APRI, AST to platelet ratio index; FIB-4, Fibrosis-4 index; SOF, sofosbuvir; RBV, ribavirin

Supplementary Table S2. Characteristics of the decompensated patients

| Patient | Gender | Age | Genotype | Treatment | Cirrhosis | Alcohol | Time after SVR (months) | HCV RNA (IU/mL) | Child-Pugh | MELD | APRI | FIB-4 |
|---|---|---|---|---|---|---|---|---|---|---|---|---|
| 1 | Male | 42 | 2a | LDV/SOF +RBV | + | - | baseline | 283,000 | 10 | 17 | 2.286 | 8.007 |
| 2 | Female | 48 | 2a | SOF+RBV | + | - | 18 HCC | 555,260 | 6 | 12 | 3.871 | 13.569 |
| 3 | Male | 76 | 2a | SOF+RBV | + | - | 33 HCC | 67,100 | 5 | 6 | 0.452 | 2.867 |
| 4 | Female | 73 | 2a | SOF+RBV | + | - | 52 | 1,260 | 6 | 11 | 1.435 | 20.954 |
| 5 | Male | 57 | 2a | SOF+RBV | + | + | baseline | 71,600 | 10 | 24 | 2.143 | 8.775 |
| 6 | Male | 60 | 1b | LDV/SOF +RBV | + | + | 6 | 673,018 | 5 | 9 | 0.727 | 4.111 |
| 7 | Female | 77 | 2 | LDV/SOF +RBV | + | - | 4 | 4,940 | 5 | 11 | 0.723 | 4.543 |
| 8 | Female | 76 | 2a | SOF+RBV | - | - | 70 | 546,000 | 5 | 6 | 0.280 | 2.450 |
| 9 | Male | 59 | 2a | SOF+RBV | + | - | baseline | 6,690 | 8 | 13 | 1.053 | 4.260 |

| | | | | | | | | | | | | |
|---|---|---|---|---|---|---|---|---|---|---|---|---|
| 10 | Female | 55 | 2 | SOF+RBV | + | + | 52 | 3,965 | 8 | 7 | 4.398 | 15.461 |
| 11 | Female | 64 | 2a | SOF+RBV | + | - | baseline | 12,700 | 6 | 7 | 0.534 | 3.653 |
| 12 | Female | 51 | 1b | LDV/SOF+RBV | + | - | baseline | 633,000 | 9 | 9 | 1.917 | 6.912 |

SVR, sustained virologic response; AFP, alpha-fetoprotein; MELD, model for end-stage liver disease; APRI, AST to platelet ratio index; FIB-4, Fibrosis-4 index; LDV, ledipasvir; SOF, sofosbuvir; RBV, ribavirin; DCV, daclatasvir
